# Supplementary material for: The Aberrant Expression of Biomarkers and Risk Prediction for Neoplastic Changes in Barrett’s Esophagus–Dysplasia
Source: Cancers (Basel). 2024 Jun 28;16(13):2386. doi: 10.3390/cancers16132386 (PMC11240336; doi:10.3390/cancers16132386)

Supplemental Figure S1.

A,1

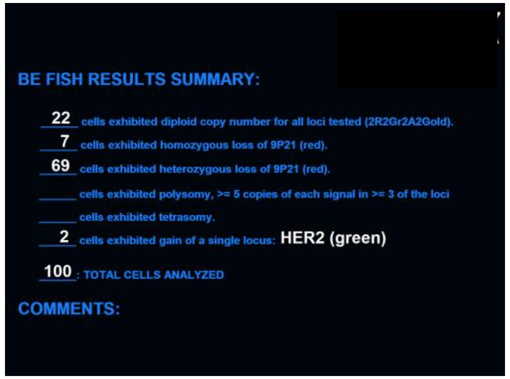
[redacted]

BE FISH RESULTS SUMMARY:

22 cells exhibited diploid copy number for all loci tested (2R2Gr2A2Gold).
7 cells exhibited homozygous loss of 9P21 (red).
69 cells exhibited heterozygous loss of 9P21 (red).
____ cells exhibited polysomy, >= 5 copies of each signal in >= 3 of the loci
____ cells exhibited tetrasomy.
2 cells exhibited gain of a single locus: HER2 (green)
100 : TOTAL CELLS ANALYZED
COMMENTS:

A,2

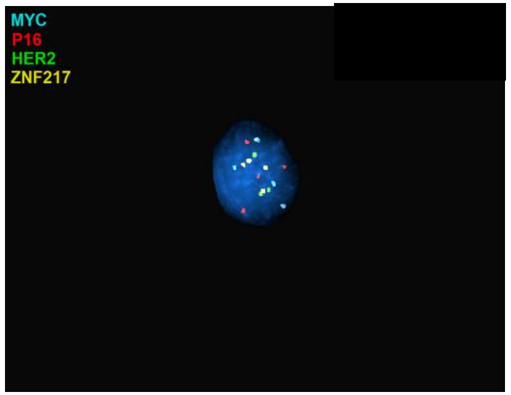

A, 3

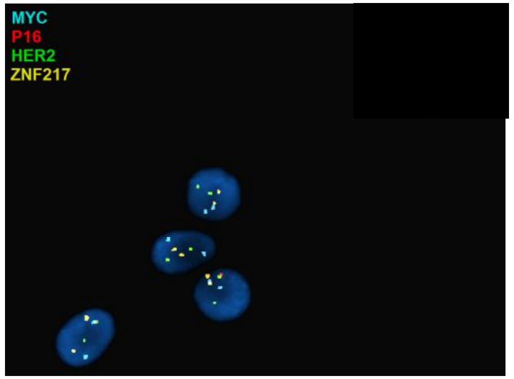

B,1

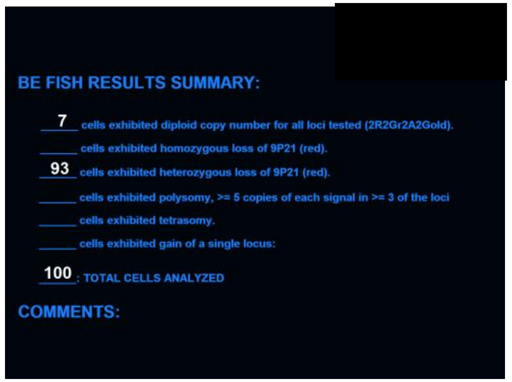
[redacted]

BE FISH RESULTS SUMMARY:

7 cells exhibited diploid copy number for all loci tested (2R2Gr2A2Gold).
____ cells exhibited homozygous loss of 9P21 (red).
93 cells exhibited heterozygous loss of 9P21 (red).
____ cells exhibited polysomy, >= 5 copies of each signal in >= 3 of the loci
____ cells exhibited tetrasomy.
____ cells exhibited gain of a single locus:
100 : TOTAL CELLS ANALYZED
COMMENTS:

B,2

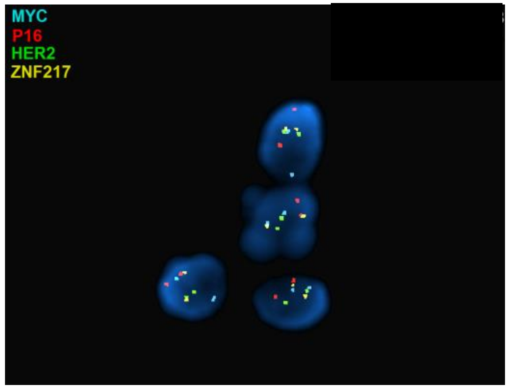

B,3

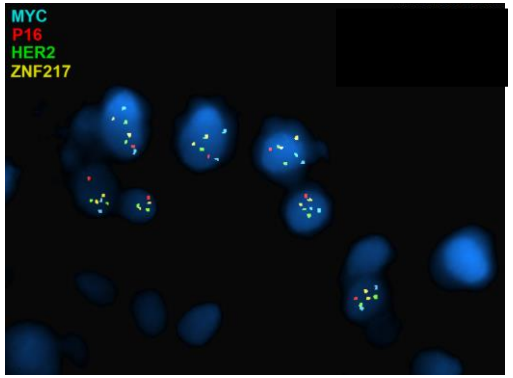

C,1

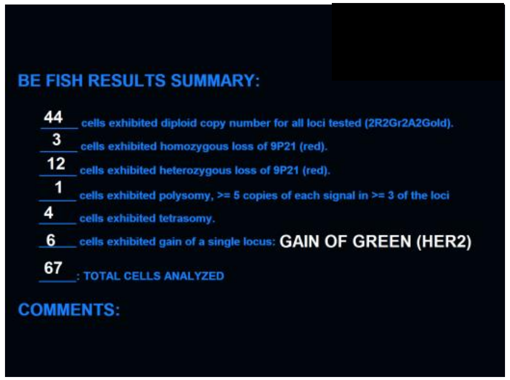
[redacted]

BE FISH RESULTS SUMMARY:

44 cells exhibited diploid copy number for all loci tested (2R2Gr2A2Gold).
3 cells exhibited homozygous loss of 9P21 (red).
12 cells exhibited heterozygous loss of 9P21 (red).
1 cells exhibited polysomy, >= 5 copies of each signal in >= 3 of the loci
4 cells exhibited tetrasomy.
6 cells exhibited gain of a single locus: GAIN OF GREEN (HER2)
67 : TOTAL CELLS ANALYZED
COMMENTS:

C,2

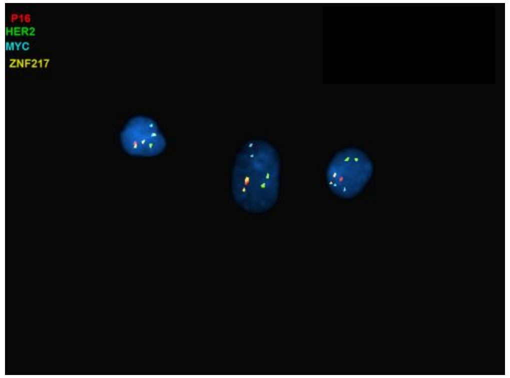

C,3

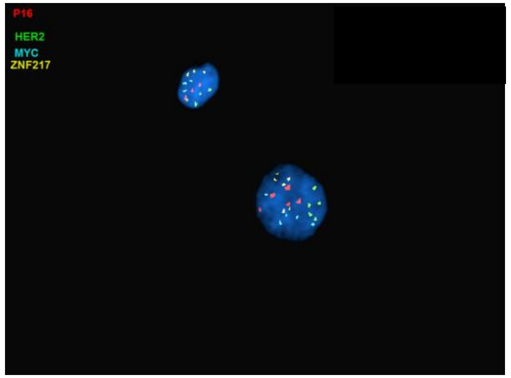

Supplement: Supplementary file 1 [file cancers-16-02386-s001.zip › cancers-3016317-supplementary.pdf]
